# Improved synthesis of structural analogues of (−)-epicatechin gallate for modulation of staphylococcal β-lactam resistance^☆^

**DOI:** 10.1016/j.tet.2014.03.052

**Published:** 2014-05-27

**Authors:** James C. Anderson, Helen Grounds, Suzanna Reeves, Peter W. Taylor

**Affiliations:** a Department of Chemistry, University College London, 20 Gordon Street, London WC1H 0AJ, UK; b School of Pharmacy, University College London, 29-39 Brunswick Square, London WC1, 1AX, UK

**Keywords:** (−)-Epicatechin gallate analogues, Asymmetric synthesis, Cyclisation, Mitsunobu, MRSA

## Abstract

The high-yielding synthesis of enantiomerically pure epicatechin gallate analogues where the A and/or B-ring hydroxylation is reduced or altered has been achieved by optimising routes to the catechin stereochemistry. The B-ring analogues were synthesised by using an electrophilic ring closure onto an enantiomerically enriched epoxide as a key step. The A and B-ring hydroxyl-deleted analogues were synthesised through a Mitsunobu cyclisation. For the B-ring analogues, the *anti*- (catechin) stereochemistry was converted to the *syn*- (epicatechin) stereochemistry by a known oxidation/reduction protocol. Absolute stereochemistry was derived from either a Sharpless epoxidation or asymmetric dihydroxylation.

## Graphical abstract

**Figure:**
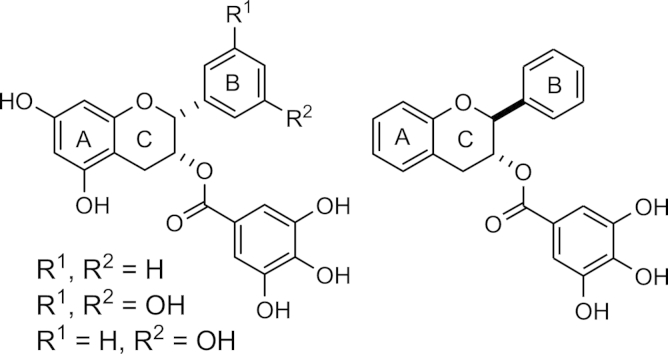

## 1 Introduction

**Fig. 1:**
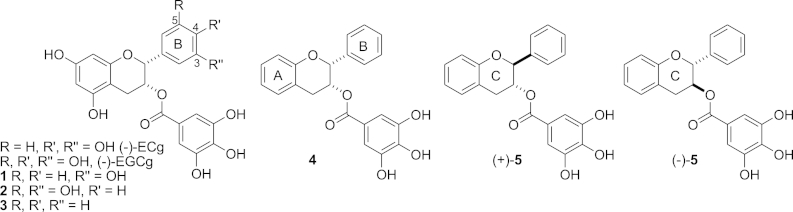
Naturally occurring galloyl catechins and non-natural targets.

Galloyl catechins, such as (−)-epicatechin gallate (ECg), (−)-epigallocatechin gallate (EGCg) and (−)-catechin gallate (Cg) are natural polyphenols, which constitute around 10% of the dry leaf weight of the green tea plant *Camellia sinensis*.^1^ They have negligible antibacterial activity themselves, but show the capacity, at relatively low concentrations, to disrupt the β-lactam resistance machinery of methicillin-resistant strains of *Staphylococcus aureus* (MRSA), inducing complete but reversible susceptibility to a wide range of β-lactam drugs.^2–4^ There is strong evidence that this effect is dependent on the intercalation of the bioactive polyphenols into the bacterial cytoplasmic membrane (CM): the most potent modifier, ECg (Fig. 1), inserts into the staphylococcal bilayer, inducing a series of complex changes to the phospholipid palisade and leading to reduction in the efficiency of function of CM-embedded proteins, such as the penicillin-binding proteins responsible for peptidoglycan biosynthesis and β-lactam resistance.^5,6^ ECg differs from EGCg only by the absence of a hydroxyl function at one of the *meta* positions on the B-ring (Fig. 1), suggesting that reducing the degree of hydroxylation or altering the position of hydroxyl groups on the B-ring pharmacophore may increase bilayer affinity, with consequent increases in bioactivity. We therefore synthesized a number of unnatural ECg analogues differing in B-ring hydroxylation and in hydroxyl substitution of the fused A–C-ring moiety (Fig. 1).^7,8^ We demonstrated that while monohydroxylated 3-hydroxy B-ring **1** and dihydroxylated 3,5-dihydroxy B-ring **2** ECg analogues sensitized MRSA strains to the β-lactam antibiotic oxacillin to a comparable extent compared to the natural product, the complete deletion of all B-ring hydroxyl groups gave a compound **3** that displayed an enhanced capacity to reduce oxacillin resistance in EMRSA-16 (Fig. 1).^8^ Complete deletion of A and B ring hydroxyl groups in either epicatechin **4** or catechin (−)-**5** resulted in a reduction in β-lactam resistance-modifying potential and an increase in intrinsic anti-staphylococcal activity, with the catechin derivative (−)-**5** showing an enhanced effect. We reported that further reduction of B-ring hydroxyl substitution led to lower synthetic yields.^8^ In order to investigate the capacity of natural and synthetic galloyl catechins, as well as combinations of galloyl and non-galloyl catechins, to interact with artificial membrane bilayers and indicate the potential for creating therapeutic catechin combinations for modulation of staphylococcal β-lactam resistance, we required a more robust synthesis of these hydroxyl deleted analogues. Here we report an improved synthesis of these analogues, as well as the synthesis of the novel enantiomer of (−)-**5** [(+)-**5**], which we have used in further studies of our own^9^ and we hope will be of use to others investigating the wide range of other biological effects exhibited by catechins in general.^10^

## 2 Results and discussion

**Scheme 1:**
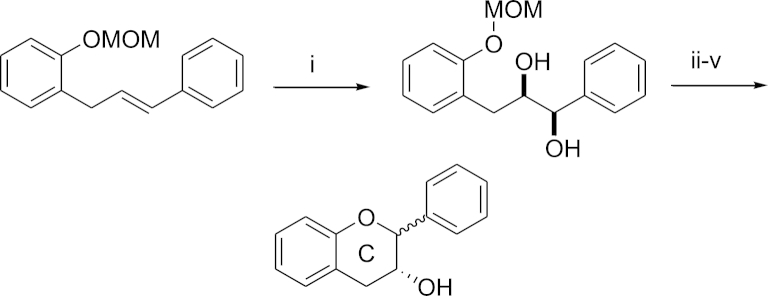
Reagents and conditions (i) AD-mix-β^®^. (ii) HCl, MeOH. (iii) HC(OMe)_3_, PPTS cat. (iv) AcBr; K_2_CO_3_. (v) NaBH_4_.

Our previous synthesis of these compounds, which was also developed by Chan,^11^ introduced the desired stereochemistry using a Sharpless dihydroxylation followed by a four-step cyclisation (Scheme 1).^7,8^ We have since found that the reproducibility of the cyclisation to form ring C is highly dependent on the purity of the acetyl bromide used. This must be freshly distilled and the presence of any HBr leads to a large mixture of often inseparable compounds, erosion of stereochemical integrity and low yields (typically less than 10%).

As 100 mg quantities of each compound were required for membrane bilayer studies we set about to develop a more reliable and reproducible synthesis of the desired analogues. Natural EGCg can be readily synthesised using a Mitsunobu cyclisation to form ring C to give the catechin followed by an oxidation and reduction to give the epicatechin.^12^ We investigated a range of conditions for the Mitsunobu cyclisations of the B-ring diminished hydroxyl compounds (**1**, **2**, **3**) along the same route, but found that in the absence of the *para*-hydroxy group on the B ring no desired product formation occurred. Since our publication of the synthesis of **2** and **3** in 2005^7^ and the synthesis of **1** and **4** in 2011^8^ a number of publications have appeared on the synthesis of various other epicatechin analogues (with or without the gallate ester), which do not have a *para*-hydroxyl group on the B-ring. A recent method replaces the capricious acetyl bromide used in our previous cyclisations with BF_3_·OEt_2_ to good effect, although only cyclises substrates to the *anti*-stereochemistry (catechin like) and in racemic form.^13^ We were drawn to the more efficient route of Yang et al. who reported the synthesis of a range of catechins using a thiourea/AuCl_3_/AgOTf catalysed diastereoselective annulation of aryl epoxides (Eq. 1).^14^ Qu et al. have reported a similar strategy for the synthesis of 3-chromanol analogues, in this case the cyclisation of a similar epoxide was performed by refluxing the substrate in hexafluoroisopropanol (HFIP), but again only on substrates, which gave the (*anti*-) catechin relative stereochemistry.^15^ They also had one example of the synthesis of a catechin analogous to **3** but with methyl ether protecting groups performed using enantiopure epoxide.(1)
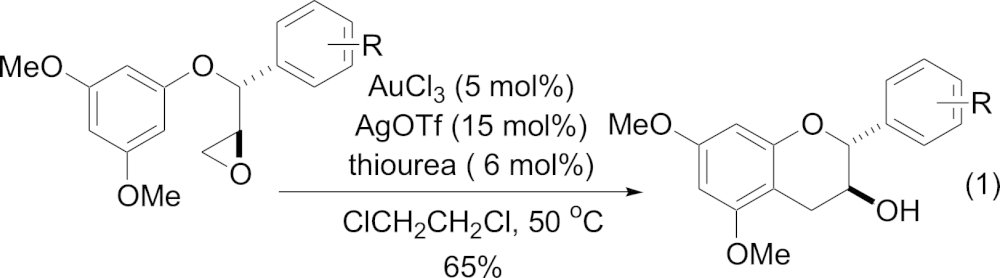


It was thought that this methodology could be applied to the synthesis of our analogues. Use of benzyl protecting groups on the phenolic hydroxyls would enable the synthesis of the ECg analogues with free phenolic hydroxyl groups after hydrogenolysis. We are not aware of any examples of the deprotection of methoxy groups on either (epi)catechins or the corresponding gallate esters. The thiourea catalyst used in Eq. 1 is not commercially available and the synthesis of this compound involves multiple steps, in addition to the use of an expensive gold catalyst. In contrast to this the HFIP cyclisation requires no additional catalyst and the solvent can be recycled and reused by a simple distillation at the end of the reaction. Although the reaction for the methoxy-substituted catechin analogous to **3** was slow (72 h—51% conversion) we decided to pursue this reaction in favour of the gold-catalysed cyclisation.

**Scheme 2:**
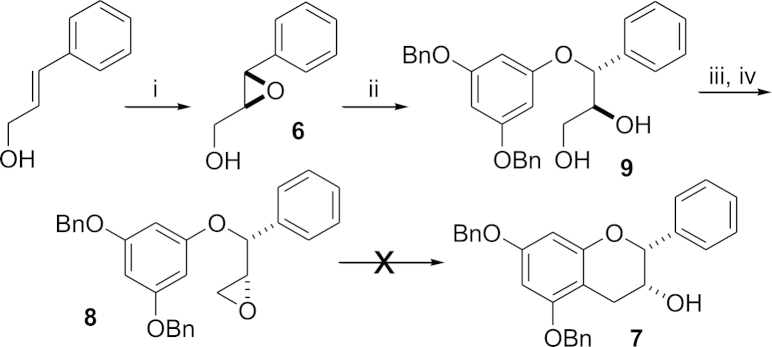
Reagents and conditions (i) mcpba, CH_2_Cl_2_, 4 h, 52%. (ii) 3,5-Dibenzoxyphenol, NaH, THF, H_2_O, reflux, 14 h, 61%. (iii) Et_3_N, TMSCl, EtOAc, −20 °C, 25 min, then MsCl, Et_3_N, 20 min followed by 2 M HCl (used crude in next step). (iv) NaOH, Bu_4_NCl, H_2_O, THF, 14 h, 73% over two steps.

For the synthesis of analogue **3** we initially attempted to synthesise the desired racemic epicatechin **7** with the correct *syn*-relative stereochemistry directly from the diastereomeric epoxide **8** compared to Yang^14^ and Qu^15^ (Scheme 2). However all attempts at cyclising **8** utilising HFIP failed, starting material and HFIP addition to the epoxide was detected in the crude reaction mixture by ^1^H NMR spectroscopy. The failure of **8** to cyclise under these reaction conditions is in direct contrast to the literature example (Fig. 1). Inspection of molecular models reveals steric crowding between each of the aromatic rings and the methylene of the epoxide in the reactive conformation required for ring closure. For the diastereoisomer in Fig. 1, the C_6_H_5_-group is orientated *exo*- to the bond forming event in the same conformation.

We therefore reverted to the diastereomeric epoxide **10a** analogous to Qu's compound.^15^ The enantiomerically pure epoxide **6a** (96% ee) was generated by Sharpless epoxidation^16^ of cinnamoyl alcohol and the epoxide synthesised for this substrate in an analogous way to epoxides synthesised by Qu. Heating **10a** in HFIP gave the desired cyclised catechin **11a** in 46% yield (56% b.r.s.m), although long reaction times were needed (15 d). The desired epicatechin relative stereochemistry was attained by oxidation of the alcohol with Dess–Martin periodinane and reduction of the generated ketone with L-Selectride to give **7a** in high overall yield.^17^ The desired gallate ester product **3** was synthesised using our previously published procedure^8^ involving the DMAP-catalysed coupling of the benzyl-protected gallate acid chloride followed by global hydrogenolysis to remove the benzyl protecting groups. By changing the solvent of the hydrogenation to a mixture of EtOAc and MeOH (compared to previously using just EtOAc) and increasing the reaction time to 12 h the yield of this step could be increased from 37 to 95% (depending on analogue) to near quantitative conversion in all cases.

**Scheme 3:**
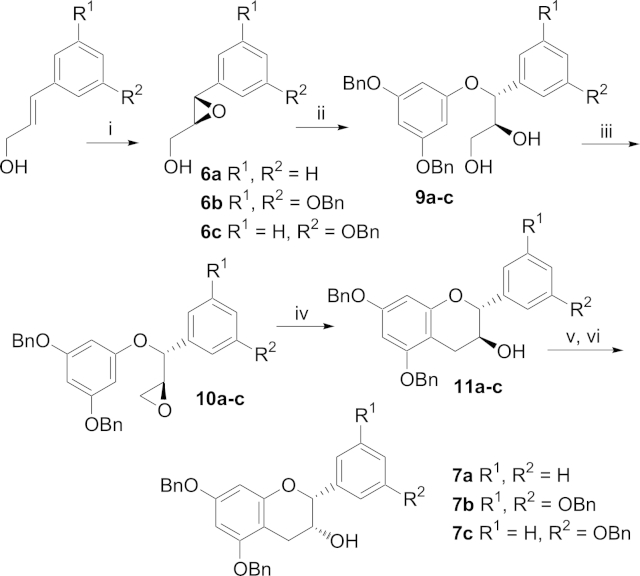
Reagents and conditions (i) Diethyl–L-tartrate, Ti(O^*i*^Pr)_4_, *t*-BuOOH, sieves, CH_2_Cl_2_, −20 °C (**6a**: 40%, **6b**: 70%, **6c**: 79%). (ii) 3,5-Dibenzoxyphenol, NaH, THF, H_2_O, reflux, 14 h (**9a**: 61%, **9b**: 61%, **9c**: 78%). (iii) **10a**: Pyridine, *p*-tosyl chloride, rt, 2 d then K_2_CO_3_ (67%); **10b**,**c**: Et_3_N, DMAP, *p*-tosyl chloride, CH_2_Cl_2_, rt, o/n then K_2_CO_3_ (**10b**: 46%, **10c**: 56%). (iv) HFIP, reflux 12–15 d (**11a**: 46%, **11b**: 42%, **11c**: 33%). (v) Dess–Martin periodinane, wet CH_2_Cl_2_, 0 °C, o/n (**a**: 63%, **b**: 57%, **c**: 68%). (vi) L-Selectride, THF, −78 °C, 2 h (**7a**: 72%, **7b**: 68%, **7c**: 74%).

This synthesis was also successful for the mono- and di-hydroxy-substituted B ring analogues (**1** and **2**). In these cases enantiopurity was determined by the formation of the Mosher ester of the epoxides **6b**,**c** as no separation was seen of the racemic epoxides using chiral HPLC. In both cases the enantiopurity was found to be >95% by ^1^H NMR analysis. The enantiopurity of all the epicatechin compounds and gallate esters was judged to be maintained through the synthetic sequence (Scheme 3) by comparison to our previously reported specific rotations.^8^

**Scheme 4:**
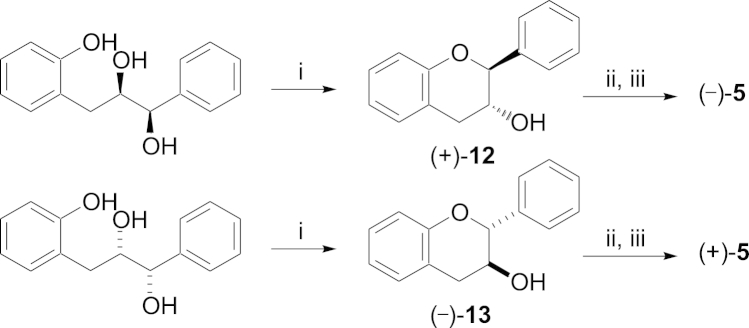
Reagents and conditions (i) PPh_3_, DEAD, THF, 14 h (**12**: 46%, **13**: 64%). (ii) BzCl, Et_3_N, DMAP (cat.), CH_2_Cl_2_, 15 h. (iii) Pd(OH)_2_/C, EtOAc/MeOH, 2.5 h ((−)-**5**: 57%), (+)-**5**: 53% over two steps).

The synthesis of our last analogues **5** was more facile. The Mitsunobu cyclisation of requisite triols has been reported by Krohn to give (−)-catechin.^18^ We repeated this reaction using both the known triols^8,18^ synthesised using α-AD mix^®^ and β-AD mix^®^ to give cyclised compounds **12** and **13**.^19^ Subsequent coupling with the benzyl-protected gallate acid chloride and hydrogenolysis gave (−)-**5** and the novel (+)-**5** (Scheme 4).

## 3 Conclusion

Reducing the degree of hydroxylation or altering the position of hydroxyl groups on the A and/or B-ring pharmacophore has a profound effect on the ability of non-natural epicatechin gallate analogues to modulate staphylococcal β-lactam resistance, and also on their syntheses. The syntheses of these molecules have proven low-yielding due to decreased activation of the aromatic rings.^7,8^ We have optimised synthetic routes to prepare these compounds in high yielding procedures, which allows for their synthesis in quantities sufficient for further studies to explore and quantify interactions with artificial membrane bilayers and indicate the potential for creating therapeutic catechin combinations for modulation of staphylococcal β-lactam resistance.^9^ The synthetic routes to the B-ring analogues rely upon formation of the *anti*- (catechin) stereochemistry, which was then converted to the *syn*- (epicatechin) stereochemistry by a known oxidation/reduction protocol.^17^ Absolute stereochemistry was derived from either a Sharpless epoxidation^16^ or asymmetric dihydroxylation.^19^ The synthetic routes will be of use to others investigating the wide range of biological effects exhibited by catechin analogues in general.

## 4 Experimental section

### 4.1 General

Melting points were recorded on a Stuart Scientific SMP3 apparatus and are uncorrected. Optical rotations were recorded on a Perkin–Elmer 343 digital polarimeter at 22 °C and are reported in deg cm^2^ g^−1^. Infrared spectra were recorded on a Perkin–Elmer 1600 FTIR instrument as thin films or solids. The NMR spectra were recorded on Bruker AVANCE III 600 MHz spectrometer as a solution in CDCl_3_ unless otherwise stated. Chemical shifts are reported in parts per million relative to CHCl_3_ (^1^H: 7.27), (^13^C: 77.2). Coupling constants are reported in Hertz and rounded to the nearest 0.1 Hz. Signal assignments are as follows: br (broad), s (singlet), d (doublet), t (triplet), q (quartet), m (multiplet), dd (doublet of doublets), etc. Mass spectra were recorded using Thermo Finnigan Mat900xp (EI/CI) and Waters LCT Premier XE (ES) instruments. HPLC analysis was acquired on a Hewlett Packard Capillary HP4890A GC analyser.

For all non-aqueous chemistry, glassware was rigorously flame-dried and an inert (N_2_) atmosphere maintained throughout. All solvents and chemicals were used as received unless stated. Chromatographic separations were performed using Merck Geduran^®^ silica gel 60. Petroleum ether with a boiling range 40–60 °C was used.

### 4.2 (±)-(*S*)-2-(*S*)-Phenyl-(3,5-dibenzyloxyphenoxy)-methyl oxirane (**8**)

A suspension of (±)-**9a** (90 mg, 0.197 mmol) and Et_3_N (30 μL, 0.197 mmol) in EtOAc (1 mL) was cooled to −20 °C and treated with a solution of TMSCl (26 μL, 0.197 mmol) in EtOAc (0.4 mL) added dropwise over 10 min. The resultant solution was stirred at −20 °C for 15 min and then treated with Et_3_N (30 μL, 0.197 mmol) followed by a solution of MsCl (17 μL, 0.217 mmol) in EtOAc (0.4 mL) and stirred for 20 min at −20 °C. After this time HCl (2 M, 0.4 mL) was added and the reaction mixture stirred at rt for 1 h. The reaction mixture was washed with H_2_O (2 mL), NaHCO_3(aq)_ (5 mL), brine (5 mL), dried (Na_2_SO_4_), filtered and concentrated in vacuo. The solid was taken up in THF (1 mL) and treated with a mixture of NaOH (35 mg, 0.450 mmol) and Bu_4_NCl (3 mg, 0.010 mmol) in H_2_O (1 mL). The resultant solution was stirred vigorously o/n. The reaction mixture was extracted into EtOAc (2×5 mL), separated, dried (MgSO_4_), filtered and concentrated in vacuo. Purification was achieved by flash column chromatography (20% EtOAc/Pet. Ether) to give **8** as a white solid (63 mg, 73%); mp 101–102 °C; *R*_*f*_ (10% EtOAc/Pet. Ether) 0.28; IR *ν*_max_ 3025, 1594, 1451, 1375, 1144, 1057 cm^−1^; ^1^H NMR *δ* 2.74 (1H, dd, *J*=4.8, 2.6, C^4^*H*_2_), 2.84 (1H, appt, *J*=4.4, C^4^*H*_2_), 3.38 (1H, ddd, *J*=6.4, 4.1, 3.3, C^3^*H*), 4.82 (1H, d, *J*=6.0, C^2^*H*), 4.94 (4H, s, 2×OC*H*_2_Ph), 6.19 (2H, d, *J*=2.4, Ar*H*), 6.21 (1H, t, *J*=2.4, Ar*H*), 7.31–7.39 (15H, m, Ar*H*); ^13^C NMR *δ* 45.1, 55.1, 70.2, 81.6, 95.4, 96.1, 126.6, 127.7, 128.1, 128.5, 128.7, 129.0, 136.8, 137.3, 159.6, 160.6; *m*/*z* (EI) 438 (M^+^), 408 (M^+^−H_2_CO), 347 (M^+^−Bn), 133, 91; HRMS *m*/*z* (EI) C_29_H_26_O_4_ calcd 438.1831, found 438.1822.

### 4.3 General procedure for the synthesis of epoxy alcohols (**6**)

A suspension of CH_2_Cl_2_ (30 mL) over molecular sieves (4 g, 4 Å, powdered) was cooled to −20 °C and treated with diethyl-l-tartrate (0.08 equiv) followed by titanium isopropoxide (0.05 equiv) and *t*-butyl hydroperoxide (2.1 equiv). The resultant solution was stirred at −20 °C for 40 min and then treated with a solution of 3,5-dibenzyloxycinnamyl alcohol^14^ (for **6b**) or 3-benzyloxycinnamyl alcohol^20^ (for **6c**) (2.47–4.16 mmol) in CH_2_Cl_2_ (20 mL), added dropwise over 1 h. After stirring for 2 h the reaction was quenched NaOH (0.21–0.33 mL, 10% aqueous solution saturated with NaCl) and diethyl ether added (6 mL). The reaction mixture was removed from the cold bath, warmed to 10 °C and stirred for 10 min after this time MgSO_4_ (0.21–0.33 g) and Celite (50 mg) were added, stirring was continued for 15 min. The reaction mixture was then filtered through Celite, washed with diethyl ether (20 mL) and concentrated in vacuo. Purification was achieved by flash column chromatography (30–50% EtOAc/Pet. Ether). Enantiopurity was checked by conversion of the alcohol to the Mosher ester. A solution of **6** (0.04 mmol) in CH_2_Cl_2_ (0.3 mL) was treated with a pre-mixed solution of Et_3_N (27 μL, 0.04 mmol) and DMAP (5 mg, 0.04 mmol) in CH_2_Cl_2_ (0.3 mL) followed by (*S*)-α-methoxy-α-(trifluoromethyl)phenylacetyl chloride (9 μL, 0.044 mmol) and stirred for 5 min, TLC analysis indicated reaction completion. The solution was concentrated and submitted for ^1^H NMR analysis (CDCl_3_). The analogous reaction was performed using racemic-3-(3-methoxy)-phenyl)-oxiran-2-yl) methanol and the distinctive double-doublets for the C^4^ peak compared.

#### 4.3.1 (2*S*,3*S*)-3-(3,5-Bis(benzyloxy)-phenyl)-oxiran-2-yl methanol (**6b**)

Yield (804 mg, 70%) as a sticky white solid; *R*_*f*_ (40% EtOAc/Pet. Ether) 0.25; [α]_D_^22^ −25.9 (*c* 1.03, CHCl_3_). ^1^H NMR data agrees with that published in the literature for the racemic compound.^14^ By comparison of the diastereotopic peaks **6b** was found to have an enantiopurity of >20:1.

#### 4.3.2 ((2*S*,3*S*)-3-(3-Benzyloxy)-phenyl)-oxiran-2-yl methanol (**6c**)

Yield (840 mg, 79%) as a white solid; mp 70–73 °C; *R*_*f*_ (30% EtOAc/Pet. Ether) 0.25; [α]_D_^22^ −34.2 (*c* 1.03, CHCl_3_); IR *ν*_max_ 3413, 3034, 2868, 1598, 1591, 1493, 1454, 1382, 1320, 1291, 1274, 1268, 1205, 1155, 1078, 1026 cm^−1^; ^1^H NMR *δ* 1.72 (1H, dd, *J*=7.9, 5.2, C^4^O*H*), 3.20 (1H, ddd, *J*=4.0, 2.2, 1.9, C^3^*H*), 3.81 (1H, ddd, *J*=12.4, 7.9, 3.7, C^4^*H*_2_), 3.92 (1H, d, *J*=1.9, C^2^*H*), 4.05 (1H, ddd, *J*=12.4, 5.2, 2.2, C^4^*H*_2_), 5.07 (2H, s, OC*H*_2_PH), 6.91–6.94 (3H, m, Ar*H*), 7.26–7.29 (1H, m, Ar*H*), 7.32–7.36 (1H, m, Ar*H*), 7.38–7.46 (4H, m, Ar*H*); ^13^C NMR *δ* 55.5, 61.2, 62.4, 70.1, 111.9, 115.0, 118.5, 127.6, 128.2, 128.7, 129.8, 136.9, 138.5, 159.2; *m*/*z* (CI) 257 (22%, M^+^+H), 239 (41%, M^+^−OH), 227 (61%), 213 (26%), 181 (26%), 161 (22%), 149 (41%, M^+^+H−OH, Bn), 137 (24%), 119 (39%), 91 (100%, Bn); HRMS C_16_H_17_O_3_ calcd 257.1172, found 257.1161. By comparison of the diastereotopic peaks **6c** was found to have an enantiopurity of >20:1.

### 4.4 General procedure for the synthesis of diols (**9**)

A solution of 3,5-dibenzoxyphenol^21^ (1.1 equiv) in anhydrous THF (6 mL) was cooled to 0 °C and treated with NaH (1.1 equiv). The resultant suspension was stirred at rt for 90 min, after this time H_2_O (9.5 mL) was added dropwise over 5 min followed by a solution of **6** (2.40–2.98 mmol, 1 equiv) in THF (6 mL). The resultant solution was heated to reflux for 1–3 d until complete by TLC. The reaction mixture was cooled to rt and concentrated in vacuo. Purification was achieved by flash column chromatography (20–70% EtOAc/Pet. Ether).

#### 4.4.1 (2*S*,3*R*)-3-(3,5-Dibenzyloxyphenoxy)-3-phenylpropane-1,2-diol (**9a**)

Yield (629 mg, 57%) as a yellow solid; mp 135–137 °C; *R*_*f*_ (40% EtOAc/Pet. Ether) 0.25; [α]_D_^22^ −8.7 (*c* 0.35, CHCl_3_); IR *ν*_max_ 3532, 3371, 1595, 1451, 1154, 1053 cm^−1^; ^1^H NMR *δ* 1.99 (1H, dd, *J*=7.4, 5.1, C^4^O*H*), 2.21 (1H, d, *J*=5.9, C^3^O*H*), 3.79–3.83 (2H, m, C^4^*H*_2_), 3.94–3.98 (1H, m, C^3^*H*), 4.93 (4H, s, 2×OC*H*_2_Ph), 5.17 (1H, d, *J*=6.0, C^2^*H*), 6.13 (2H, d, *J*=2.1, Ar*H*), 6.20 (1H, t, *J*=2.1, Ar*H*), 7.30–7.41 (15H, m, Ar*H*); ^13^C NMR *δ* 62.9, 70.2, 74.8, 81.1, 95.4, 96.0, 126.8, 127.7, 128.2, 128.5, 128.7, 129.0, 136.7, 137.6, 159.4, 160.6; *m*/*z* (CI) 457 (53%, M^+^+H), 439 (23%), 307 (100%); HRMS *m*/*z* (CI) C_29_H_29_O_5_ calcd 457.2015, found 457.2003.

#### 4.4.2 (2*S*,3*R*)-3-(3,5-Dibenzyloxyphenoxy)-3-(3,5-dibenzyloxyphenyl)-propane-1,2-diol (**9b**)

Yield (919 mg, 61%) as a white solid; mp 66–68 °C; *R*_*f*_ (40% EtOAc/Pet. Ether) 0.20; [α]_D_^22^ −2.3 (*c* 1.10, CHCl_3_); IR *ν*_max_ 3403, 1597, 1454, 1377, 1156, 1056 cm^−1^; ^1^H NMR *δ* 2.00–2.02 (1H, m, C^4^O*H*), 2.50 (1H, d, *J*=5.9, C^3^O*H*), 3.73–3.81 (2H, m, C^4^*H*_2_), 3.89–3.94 (1H, m, C^3^*H*), 4.94 (4H, s, 2×OC*H*_2_Ph), 5.01 (4H, s, 2×OC*H*_2_Ph), 5.07 (1H, d, *J*=5.9, C^2^*H*), 6.14 (1H, s, Ar*H*), 6.14 (1H, s, Ar*H*), 6.23 (1H, br, Ar*H*), 6.57 (1H, br, Ar*H*), 6.61 (1H, s, Ar*H*), 6.62 (1H, s, Ar*H*), 7.27–7.41 (20H, m, Ar*H*); ^13^C NMR *δ* 62.9, 70.2, 70.3, 74.7, 81.0, 95.4, 96.0, 101.9, 105.8, 127.7, 127.8, 128.21, 128.24, 128.69, 128.72, 136.6, 136.8, 140.1, 159.4, 160.4, 160.6; *m*/*z* (CI) 669 (62%, M^+^), 363 (28%, M^+^−3×Bn, OH_2_), 352 (82%), 207 (100%), 181 (25%), 91 (26%, Bn); HRMS *m*/*z* (ES) C_43_H_39_O_7_ calcd 667.2696, found 667.2755.

#### 4.4.3 (2*S*,3*R*)-3-(3,5-Dibenzyloxyphenoxy)-3-(3-benzyloxyphenyl)-propane-1,2-diol (**9c**)

Yield (1.30 g, 78%) as a white solid; mp 78–80 °C; *R*_*f*_ (30% EtOAc/Pet. Ether) 0.25; [α]_D_^22^ −14.8 (*c* 1.14, CHCl_3_); IR *ν*_max_ 3406, 1594, 1450, 1378, 1260, 1154, 1060 cm^−1^; ^1^H NMR *δ* 2.00–2.02 (1H, m, C^4^O*H*), 2.23 (1H, d, *J*=5.4, C^3^O*H*), 3.75–3.81 (2H, m, C^4^*H*_2_), 3.91–3.94 (1H, m, C^3^*H*), 4.92 (4H, s, 2×OC*H*_2_Ph), 5.03 (2H, s, OC*H*_2_Ph), 5.11 (1H, d, *J*=5.9, C^2^*H*), 6.13 (1H, s, Ar*H*), 6.13 (1H, s, Ar*H*), 6.91 (1H, dd, *J*=8.1, 1.7, Ar*H*), 6.94 (1H, d, *J*=7.6, Ar*H*), 6.98 (1H, s, Ar*H*), 7.26–7.42 (17H, m, Ar*H*); ^13^C NMR *δ* 62.9, 70.1, 70.2, 74.8, 80.9, 95.4, 96.0, 113.2, 114.8, 119.4, 127.71, 127.74, 128.18, 128.22, 128.71, 128.73, 130.1, 136.8 (×2), 139.3, 159.3, 159.4, 160.6; *m*/*z* (CI) 563 (16%, M^+^+H), 545 (10%, M^+^−OH), 338 (10%), 308 (21%), 307 (100%), 306 (12%), 239 (17%), 223 (13%); HRMS C_36_H_35_O_6_ calcd 563.2428, found 563.2427.

### 4.5 (*S*)-2-(*R*)-Phenyl-(3,5-dibenzyloxyphenoxy)-methyl oxirane (**10a**)

A solution of **9a** (450 mg, 0.980 mmol) in pyridine (2.5 mL) was cooled to 0 °C and treated with *p*-tosyl chloride (186 mg, 0.98 mmol). The resultant reaction mixture was stirred at rt for 2 d. 1 M HCl_(aq)_ (20 mL) was added to the reaction mixture and the solution was extracted wit EtOAc (3×15 mL). The organics were washed with brine (2×20 mL), NaHCO_3(aq)_ solution (30% wt, 2×20 mL), brine (2×15 mL), separated, dried (Na_2_SO_4_), filtered and concentrated in vacuo. The residue was taken up in MeOH (8 mL), treated with K_2_CO_3_ (145 mg, 1.40 mmol) and stirred at rt o/n. After this time H_2_O (20 mL) was added and the solution extracted Et_2_O (3×20 mL), washed with brine (3×20 mL), dried (Na_2_SO_4_), filtered and concentrated in vacuo. Purification was achieved by flash column chromatography (10–50% EtOAc/Pet. Ether) to give **10a** (289 mg, 67%, (90% b.r.s.m.)) as a pale yellow solid; mp 90–93 °C; *R*_*f*_ (30% EtOAc/Pet. Ether) 0.64; [α]_D_^22^ −2.8 (*c* 1.16, CHCl_3_); IR *ν*_max_ 1595, 1450, 1378, 1260, 1153, 1060 cm^−1^; ^1^H NMR *δ* 2.79 (1H, dd, *J*=5.2, 2.5, C^4^*H*_2_), 2.83 (1H, dd, *J*=5.0, 4.0, C^4^*H*_2_), 3.32 (1H, dt, *J*=4.0, 4.0, C^3^*H*), 4.94 (4H, s, 2×OC*H*_2_Ph), 5.08 (1H, d, *J*=4.1, C^2^*H*), 6.16 (2H, d, *J*=2.1, Ar*H*), 6.21 (1H, t, *J*=2.1, Ar*H*), 7.27–7.40 (15H, m, Ar*H*); ^13^C NMR *δ* 45.1, 54.4, 70.2, 79.2, 95.4, 96.2, 126.8, 127.7, 128.1, 128.5, 128.7, 128.8, 136.8, 137.4, 159.5, 160.6; *m*/*z* (CI) 439 (100%, M^+^), 414 (71%), 240 (22%), 229 (42%), 87 (36%), 85 (75%); HRMS C_29_H_27_O_4_ calcd 439.1909, found 439.1893.

### 4.6 General procedure for the synthesis of epoxides (**10**)

A solution of **9** (1.33–1.42 mmol) in CH_2_Cl_2_ (30 mL) was treated with Et_3_N (1.5 equiv), DMAP (0.025 equiv) and *p*-tosyl chloride (1.2 equiv). The resultant reaction mixture was stirred at rt o/n. The reaction mixture was washed with H_2_O (20 mL), separated, dried (Na_2_SO_4_), filtered and concentrated in vacuo. The residue was taken up in MeOH (50 mL), treated with K_2_CO_3_ (1.3 equiv) and stirred at rt for 2 h. The solvent was removed in vacuo and the residue taken up in EtOAc (40 mL), washed with H_2_O (20 mL), dried (MgSO_4_), filtered and concentrated in vacuo. Purification was achieved by flash column chromatography (10–50% EtOAc/Pet. Ether).

#### 4.6.1 (*S*)-2-(*R*)-(3,5-Bis(benzyloxy)-phenyl)-(3,5-dibenzyloxyphenoxy)-methyl oxirane (**10b**)

Yield (402 mg, 46%, (58% b.r.s.m.)) as a white solid; mp 64–66 °C; *R*_*f*_ (30% EtOAc/Pet. Ether) 0.46; [α]_D_^20^ −16.1 (*c* 1.30, CHCl_3_); IR *ν*_max_ 3063, 2864, 1594, 1444, 1379, 1354, 1290, 1158, 1080, 1056, 1028 cm^−1^; ^1^H NMR *δ* 2.80–2.82 (2H, m, C^4^*H*_2_), 3.27–3.29 (1H, dt, *J*=3.6, 3.2, C^3^*H*), 4.95 (4H, s, 2×OC*H*_2_Ph), 4.99 (1H, d, *J*=4.0, C^2^*H*), 5.02 (4H, s, 2×OC*H*_2_Ph), 6.16 (2H, d, *J*=1.9, Ar*H*), 6.23 (1H, t, *J*=1.9, Ar*H*), 6.58 (1H, t, *J*=1.9, Ar*H*), 6.67 (2H, d, *J*=1.9, Ar*H*), 7.31–7.43 (20H, m, Ar*H*); ^13^C NMR *δ* 45.1, 54.5, 70.2, 70.3, 79.1, 95.5, 96.1, 101.9, 105.8, 127.2, 127.3, 127.8, 128.5, 128.9, 137.2, 143.0, 159.6, 160.3, 160.6; *m*/*z* (CI) 651 (100%, M^+^+H), 621 (15%), 414 (16%), 345 (21%), 307 (23%), 181 (17%); HRMS *m*/*z* (CI) C_43_H_39_O_6_ calcd 651.2747, found 651.2739.

#### 4.6.2 (*S*)-2-(*R*)-(3-Benzyloxyphenyl)-(3,5-dibenzyloxyphenoxy)-methyl oxirane (**10c**)

Yield (433 mg, 56%, (81% b.r.s.m.)) as a waxy pale yellow solid; *R*_*f*_ (25% EtOAc/Pet. Ether) 0.60; [α]_D_^22^ −12.1 (*c* 0.73, CHCl_3_); IR *ν*_max_ 3032, 1598, 1454, 1379, 1262, 1156, 1056 cm^−1^; ^1^H NMR *δ* 2.76–2.81 (2H, m, C^4^*H*_2_), 3.27–3.30 (1H, m, C^3^*H*), 4.93 (4H, s, 2×OC*H*_2_Ph), 5.04 (3H, s, C^2^*H*, OC*H*_2_Ph), 6.15 (2H, d, *J*=2.1, Ar*H*), 6.21 (1H, t, *J*=2.1, Ar*H*), 6.92 (1H, dd, *J*=8.1, 2.2, Ar*H*), 6.98 (1H, d, *J*=7.7, Ar*H*), 7.02 (1H, m, Ar*H*), 7.27–7.48 (16H, m, Ar*H*); ^13^C NMR *δ* 45.1, 54.4, 70.1, 70.2, 79.0, 95.4, 96.1, 113.2, 114.8, 119.4, 127.7, 127.8, 128.1, 128.7, 129.9, 136.8, 136.9, 139.1, 159.2, 159.5, 160.6; *m*/*z* (EI) 544 (6%, M^+^), 149 (30%), 123 (24%), 111 (31%), 97 (52%), 83 (53%), 81 (61%), 69 (100%); HRMS C_36_H_32_O_5_ calcd 544.2244, found 544.2248.

### 4.7 General procedure for the synthesis of catechins (**11**)

A solution of **10** (0.691–0.958 mmol) in HFIP (15 mL) was heated to reflux in a sealed tube for 12–15 d. After this time the solvent was distilled off and the residue purified by flash column chromatography (5–25 % Et_2_O/Pet. Ether).

#### 4.7.1 (2*R*,3*S*)-2-Phenyl-5,7-dibenzyloxy-chroman-3-ol (**11a**)

Yield (203 mg, 46%, 56% b.r.s.m.) as a white solid; mp 134–135 °C; *R*_*f*_ (30% EtOAc/Pet. Ether) 0.50; [α]_D_^22^ +10.2 (*c* 0.29, CH_2_Cl_2_), (lit.^5^ (other enantiomer) no mp reported, [α]_D_ −8.7 (*c* 0.30, CH_2_Cl_2_)). ^1^H NMR data agrees with those published in the literature for the other enantiomer.^8^

#### 4.7.2 (2*R*,3*S*)-2-(3,5-Bis-benzyloxy-phenyl)-5,7-dibenzyloxy-chroman-3-ol (**11b**)

Yield (167 mg, 42%, 74% b.r.s.m.) as a white solid; mp 100–102 °C; *R*_*f*_ (25% EtOAc/Pet. Ether) 0.50; [α]_D_^22^ −2.0 (*c* 1.04, CHCl_3_); IR *ν*_max_ 3567, 3064, 3032, 2916, 1594, 1498, 1455, 1375, 1346, 1291, 1216, 1150, 1118, 1053, 1029 cm^−1^; ^1^H NMR *δ* 1.72 (1H, d, *J*=3.6, C^3^O*H*), 2.67 (1H, dd, *J*=16.4, 8.8, C^4^*H*_2_), 3.10 (1H, dd, *J*=16.4, 5.6, C^4^*H*_2_), 4.03–4.90 (1H, m, C^3^*H*), 4.69 (1H, d, *J*=8.0, C^2^*H*), 5.00 (2H, s, OC*H*_2_Ph), 5.03 (4H, s, 2×OC*H*_2_Ph), 5.04 (2H, s, OC*H*_2_Ph), 6.24 (1H, d, *J*=2.2, Ar*H*), 6.27 (1H, d, *J*=2.2, Ar*H*), 6.61 (1H, t, *J*=2.2, Ar*H*), 6.69 (2H, d, *J*=2.2, Ar*H*), 7.32–7.39 (20H, m, Ar*H*); ^13^C NMR *δ* 27.6, 68.3, 70.0, 70.2, 70.3, 77.3, 81.8, 93.9, 94.4, 102.2, 102.4, 106.3, 127.3, 127.7, 127.7, 128.0, 128.1, 128.2, 128.6, 128.7, 128.7, 136.7, 136.9, 137.0, 140.4, 155.2, 157.9, 158.9, 160.4; *m*/*z* (EI) 650 (20%, M^+^−H), 369 (32%), 355 (21%), 319 (17%), 242 (16%), 91 (100%, Bn); HRMS *m*/*z* (EI) C_43_H_38_O_6_ calcd 650.2663, found 650.2666.

#### 4.7.3 (2*R*,3*S*)-2-(3-Benzyloxyphenyl)-5,7-dibenzyloxy-chroman-3-ol (**11c**)

Yield (150 mg, 33%, 71% b.r.s.m.) as a white solid; mp 86–88 °C; *R*_*f*_ (25% EtOAc/Pet. Ether) 0.47; [α]_D_^22^ −4.0 (*c* 0.98, CHCl_3_); IR *ν*_max_ 3417, 3032, 2907, 1617, 1592, 1496, 1441, 1377, 1289, 1218, 1150, 1118, 1049, 1029 cm^−1^; ^1^H NMR *δ* 1.72 (1H, s, C^3^O*H*), 2.69 (1H, dd, *J*=16.4, 8.6, C^4^*H*_2_), 3.10 (1H, dd, *J*=16.4, 5.6, C^4^*H*_2_), 4.09–4.10 (1H, m, C^3^*H*), 4.75 (1H, d, *J*=7.9, C^2^*H*), 5.00 (2H, s, OC*H*_2_Ph), 5.03 (2H, s, OC*H*_2_Ph), 5.07 (2H, s, OC*H*_2_Ph), 6.24 (1H, d, *J*=2.2, Ar*H*), 6.28 (1H, d, *J*=2.2, Ar*H*), 6.97 (1H, dd, *J*=8.3, 2.3, Ar*H*), 7.03 (1H, d, *J*=7.7, Ar*H*), 7.07 (1H, m, Ar*H*), 7.31–7.40 (16H, m, Ar*H*); ^13^C NMR *δ* 27.6, 68.3, 70.0, 70.1, 70.2, 77.3, 81.8, 93.9, 94.4, 102.2, 113.6, 115.2, 119.8, 127.3, 127.7, 127.7, 128.0, 128.1, 128.2, 128.7, 128.7, 128.7, 130.1, 136.8, 137.0, 137.0, 139.7, 155.3, 157.9, 158.9, 159.2; *m*/*z* (CI) 545 (100%, M^+^−H), 545 (18%, M^+^), 527 (16%), 319 (16%); HRMS *m*/*z* (CI) C_36_H_32_O_5_ calcd 545.2323, found 545.2328.

### 4.8 General procedure for the synthesis of epicatechins (**7**)

*Step 1*: A solution of **11** (0.150–0.460 mmol) in wet CH_2_Cl_2_ (3–15 mL) was cooled to 0 °C and treated with Dess–Martin periodinane (1.2 equiv). The resultant solution was stirred at rt o/n. The reaction mixture was washed with 1 M NaOH_(aq)_, back extracted with CH_2_Cl_2_, organics combined and washed with brine, dried (MgSO_4_), filtered and concentrated in vacuo. Purification was achieved by flash column chromatography (10% EtOAc/Pet. Ether).

*Step 2*: A solution of ketone (0.142–0.330 mmol) in anhydrous THF (5 mL) was cooled to −78 °C and treated with L-Selectride (1.4 equiv, 1 M in THF). The resultant solution was stirred at −78 °C for 2 h. The reaction mixture was taken up in EtOAc (10 mL) washed with H_2_O (5 mL), back extracted with EtOAc (10 mL), organics combined and dried (Na_2_SO_4_), filtered and concentrated in vacuo. Purification was achieved by flash column chromatography (15% EtOAc/Pet. Ether).

#### 4.8.1 (2*R*,3*R*)-2-Phenyl-5,7-dibenzyloxy-chroman-3-ol^7^ (**7a**)

##### 4.8.1.1 Step 1: (2*R*)-2-(3,5-bis-benzyloxy-phenyl)-5,7-dibenzyloxy-chroman-3-on

Yield (125 mg, 63%) as a white solid; mp 109–111 °C; *R*_*f*_ (30% EtOAc/Pet. Ether) 0.75; [α]_D_^22^ +16.5 (*c* 0.74, CHCl_3_); IR *ν*_max_ 2919, 2872, 1731, 1594, 1451, 1378, 1152, 1027 cm^−1^; ^1^H NMR *δ* 3.51 (1H, d, *J*=22.4, C^4^*H*_2_), 3.67 (1H, d, *J*=22.4, C^4^*H*_2_), 5.02 (2H, s, OC*H*_2_Ph), 5.05 (2H, s, OC*H*_2_Ph), 5.36 (1H, s, C^2^*H*), 6.36 (1H, d, *J*=2.2, Ar*H*), 6.41 (1H, d, *J*=2.1, Ar*H*), 7.33–7.45 (15H, m, Ar*H*); ^13^C NMR *δ* 33.9, 70.2, 70.4, 83.6, 95.2, 95.9, 102.0, 126.8, 127.3, 127.7, 128.2, 128.3, 128.7, 128.7, 128.8, 128.8, 135.2, 136.6, 136.7, 154.7, 157.2, 159.6, 205.2; *m*/*z* (CI) 437 (100%, M^+^+H), 414 (25%), 219 (20%), 181 (22%), 91 (85%, Bn); HRMS *m*/*z* (CI) C_29_H_25_O_4_ calcd 437.1753, found 437.1749.

##### 4.8.1.2 Step 2: (2*R*,3*R*)-2-phenyl-5,7-dibenzyloxy-chroman-3-ol^7^

Yield (91 mg, 72%) as a waxy solid; *R*_*f*_ (25% Et_2_O/Pet. Ether) 0.21; [α]_D_^22^ −11.0 (*c* 0.45, CH_2_Cl_3_), (lit.^7^ [α]_D_^20^ −10.5 (*c* 0.40, CH_2_Cl_2_)). ^1^H NMR data agrees with those published in the literature.^7^

#### 4.8.2 (2*R*,3*R*)-2-(3,5-Bis-benzyloxy-phenyl)-5,7-dibenzyloxy-chroman-3-ol^7^ (**7b**)

##### 4.8.2.1 Step 1: (2*R*)-2-(3,5-bis-benzyloxy-phenyl)-5,7-dibenzyloxy-chroman-3-one

Yield (94 mg, 57%, (73% b.r.s.m.)) as a white solid; mp 112–114 °C; *R*_*f*_ (10% EtOAc/Pet. Ether) 0.34; [α]_D_^22^ +24.6 (*c* 1.06, CHCl_3_); IR *ν*_max_ 3032, 2919, 2872, 1732, 1619, 1595, 1498, 1453, 1441, 1376, 1348, 1293, 1217, 1179, 1152, 1099, 1081, 1052, 1029 cm^−1^; ^1^H NMR *δ* 3.49 (1H, d, *J*=21.4, C^4^*H*_2_), 3.63 (1H, d, *J*=21.4, C^4^*H*_2_), 4.99 (4H, s, 2×OC*H*_2_Ph), 5.02 (2H, s, OC*H*_2_Ph), 5.04 (2H, s, OC*H*_2_Ph), 5.29 (1H, s, C^2^*H*), 6.35 (1H, d, *J*=2.0, Ar*H*), 6.40 (1H, d, *J*=1.9, Ar*H*), 6.58 (1H, t, *J*=1.9, Ar*H*), 6.64 (2H, d, *J*=2.0, Ar*H*), 7.32–7.40 (20H, m, Ar*H*); ^13^C NMR *δ* 33.8, 70.2, 70.2, 70.4, 83.3, 95.2, 95.8, 101.9, 102.3, 105.8, 127.4, 127.7, 127.8, 128.2, 128.3, 128.7, 128.8, 136.6, 136.7, 137.4, 154.5, 157.2, 159.6, 160.2, 204.8; *m*/*z* (CI) 649 (4%, M^+^+H), 614 (28%), 414 (100%), 219 (74%), 91 (86%, Bn); HRMS *m*/*z* (CI) C_43_H_37_O_6_ calcd 649.2590, found 649.2589.

##### 4.8.2.2 Step 2: (2*R*,3*R*)-2-(3,5-bis-benzyloxy-phenyl)-5,7-dibenzyloxy-chroman-3-ol^7^

Yield (63 mg, 68%) as a white solid; mp 121–123 °C; *R*_*f*_ (20% EtOAc/Pet. Ether) 0.64; [α]_D_^22^ −17.8 (*c* 0.8, CH_2_Cl_2_), (lit.^7^ [α]_D_ −17.2 (*c* 0.8, CH_2_Cl_2_, no mp reported)). ^1^H NMR data agrees with literature data.^7^

#### 4.8.3 (2*R*,3*R*)-2-(3-Benzyloxy-phenyl)-5,7-dibenzyloxy-chroman-3-ol^7^ (**7c**)

##### 4.8.3.1 Step 1: (2*R*)-2-(3-benzyloxyphenyl)-5,7-dibenzyloxy-chroman-3-one

Yield (36 mg, 44%, 68% b.r.s.m.) as a white solid; mp 100–102 °C; *R*_*f*_ (10% EtOAc/Pet. Ether) 0.36; [α]_D_^22^ +17.4 (*c* 1.25, CHCl_3_); IR *ν*_max_ 2921, 1733, 1620, 1595, 1498, 1454, 1441, 1377, 1293, 1217, 1179, 1152, 1099, 1081, 1052, 1029 cm^−1^; ^1^H NMR *δ* 3.49 (1H, d, *J*=21.4, C^4^*H*_2_), 3.65 (1H, d, *J*=21.4, C^4^*H*_2_), 5.01 (2H, s, OC*H*_2_Ph), 5.02 (2H, s, OC*H*_2_Ph), 5.04 (2H, s, OC*H*_2_Ph), 5.33 (1H, s, C^2^*H*), 6.35 (1H, d, *J*=2.2, Ar*H*), 6.40 (1H, d, *J*=2.1, Ar*H*), 6.94 (1H, dd, *J*=8.2, 2.3, Ar*H*), 6.94 (1H, d, *J*=7.8, Ar*H*), 7.01 (1H, m, Ar*H*), 7.26–7.48 (16H, m, Ar*H*); ^13^C NMR *δ* 33.8, 70.1, 70.2, 70.3, 83.3, 95.2, 95.9, 113.2, 115.1, 119.3, 127.3, 127.7, 128.2, 128.2, 128.3, 128.7, 128.8, 129.9, 136.6, 136.7, 136.8, 154.6, 157.2, 159.1, 159.6, 205.0; *m*/*z* (CI) 543 (26%, M^+^+H), 414 (100%), 219 (72%), 111 (16%); HRMS C_36_H_31_O_5_ calcd 543.2172, found 543.2175.

##### 4.8.3.2 Step 2: (2*R*,3*R*)-2-(3-benzyloxy-phenyl)-5,7-dibenzyloxy-chroman-3-ol^7^

Yield (66 mg, 74%) as a white solid; mp 112–114 °C; *R*_*f*_ (20% EtOAc/Pet. Ether) 0.58; [α]_D_^22^ −28.1 (*c* 1.06, CH_2_Cl_2_), (lit.^7^ [α]_D_ −25.7 (*c* 3.4, CH_2_Cl_2_, no mp reported)). ^1^H NMR data agrees with literature data.^7^

### 4.9 Synthesis of (2*S*,3*R*)-*trans*-flavan-3-ol (**12**)^8^

Synthesised according to the procedure of Krohn et al. using (1*R*,2*R*)-3-(2-hydroxyphenyl)-1-phenylpropane-1,2-diol in place of (1*S*,2*S*)-3-(2-hydroxyphenyl)-1-phenylpropane-1,2-diol to give the opposite enantiomer.^18^ Yield (88 mg, 46%, (66% b.r.s.m)) as a white solid; mp 70–72 °C; [α]_D_^20^ +8.51 (*c* 1.32, CH_2_Cl_2_), (lit. mp and [α]_D_ not reported). ^1^H NMR agrees with literature data for racemic material.^22^

### 4.10 Synthesis of hydroxyl deleted A and B ring catechin gallates (**5**)

#### 4.10.1 (+)-(2*R*,3*S*)-*trans*-Phenylflavan-3-yl-3,4,5-tris(benzyloxy)benzoate

Synthesised according to the procedure of Anderson et al. using (2*S*,3*R*)-*trans*-flavan-3-ol in place of (2*R*,3*S*)-*trans*-flavan-3-ol to give the opposite enantiomer.^8^ Yield (165 mg, 62%) as a white solid; mp 121–123 °C; [α]_D_^20^ +60.5 (*c* 0.50, CH_2_Cl_2_), (lit.^8^ mp 126–128 °C, [α]_D_^20^ −65.0 (*c* 0.50, CH_2_Cl_2_) opposite enantiomer). ^1^H NMR agrees with literature data for opposite enantiomer.^8^

#### 4.10.2 (+)-(2*R*,3*S*)-*trans*-Phenylflavan-3-yl-*O*-gallate (**5**)

A solution of (+)-(2*R*,3*S*)-*trans*-phenylflavan-3-yl-3,4,5-tris(benzyloxy)benzoate (55 mg, 0.084 mmol) in EtOAc (10 mL) and MeOH (10 mL) was treated with Pd(OH)_2_/C (42 mg) and back filled with H_2_ (three times). Stirred at rt for 2.5 h, filtered through Celite, washed with EtOAc (10 mL) and concentrated to give **5** (27 mg, 85%) as an off white solid; mp 210–212 °C; [α]_D_^20^ +95.1 (*c* 0.30, acetone), (lit.^8^ mp not reported, [α]_D_^20^ −97.0 (*c* 0.3, acetone) opposite enantiomer). ^1^H NMR agrees with literature data for opposite enantiomer.^8^
